# IL-2 augments the therapeutic efficacy of adoptively transferred B cells which directly kill tumor cells via the CXCR4/CXCL12 and perforin pathways

**DOI:** 10.18632/oncotarget.11124

**Published:** 2016-08-09

**Authors:** Yang Xia, Huimin Tao, Yangyang Hu, Quanning Chen, Xin Chen, Leiming Xia, Li Zhou, Yi Wang, Yangyi Bao, Shiang Huang, Xiubao Ren, Steven K. Lundy, Fu Dai, Qiao Li, Alfred E. Chang

**Affiliations:** ^1^ Department of Surgery, University of Michigan, Ann Arbor, Michigan, USA; ^2^ The No.1 People's Hospital of Hefei, Hefei, China; ^3^ Hubei Province Stem Cell Research & Appling Center, Wuhan Union Hospital, Wuhan, China; ^4^ Department of General Surgery, Tongji Hospital of Tongji University, Shanghai, China; ^5^ Department of Oncology, Wuhan University, Renmin Hospital, Wuhan, China; ^6^ Department of Biotherapy, Tianjin University Cancer Institute and Hospital, National Clinical Research Center of Cancer, Key laboratory of Cancer Immunology and Biotherapy, Tianjin, China; ^7^ Department of Internal Medicine, University of Michigan, Ann Arbor, Michigan, USA; ^8^ Current address: Fuda Cancer Hospital, Jinan University School of Medicine and Fuda Cancer Institute, Guangzhou, China

**Keywords:** B cells, adoptive immunotherapy, IL-2, CXCR4/CXCL12, perforin

## Abstract

We previously reported that antitumor B cells directly kill tumor cells *via* the Fas/FasL pathway and are regulated by IL-10. In this study, we defined additional mechanisms involved in B cell antitumor immunity. Administration of IL-2 significantly augmented the therapeutic efficacy of adoptively transferred tumor-draining lymph node (TDLN) B cells which express IL- 2R. Culture supernatant of purified B splenocytes harvested from the mice that received adoptive transfer of 4T1 TDLN B cells plus IL-2 administration produced larger amounts of IgG which bound to 4T1, resulting in 4T1 lysis. Furthermore, we detected CXCR4 expression on 4T1 TDLN B cells, and 4T1 tumor cells produced its ligand CXCL12. Transwell experiments demonstrated the chemoattraction of CXCR4-expressing 4T1 TDLN B cells towards CXCL12- producing 4T1 cells. Blockade of CXCR4 using a CXCR4-specific inhibitor, AMD3100, significantly reduced the killing of 4T1 tumor cells by 4T1 TDLN B cells. Blockade of FasL and CXCR4 concurrently inhibited B cell-mediated direct killing of tumor cells in an additive manner, indicating that both Fas/FasL and CXCL12/CXCR4 pathways are involved in the direct killing of 4T1 cells by 4T1 TDLN B cells. TDLN B cells produced perforin. Additional transwell experiments showed that effector B cells could directly kill tumor cells in cell-cell contact *via* the Fas/FasL and CXCR4/CXCL12 pathways as well as perforin, while without cell contact, perforin secreted by B cells led to tumor cell cytotoxicity. These findings underscore the diversity of function by which B cells can play an important role in the host immune response to tumor.

## INTRODUCTION

We previously reported that simultaneously stimulating CD3 on T cells and CD40 on B cells augments the antitumor reactivity of tumor-draining lymph node (TDLN) cells [1]. Furthermore, we demonstrated that *in vivo* sensitized and *in vitro* activated TDLN B cells mediate tumor regression in cancer adoptive immunotherapy [2]. In hosts that received whole body irradiation to delete lymphoid cells, the subsequent transfer of activated B cells had significant antitumor effects on established tumors [2]. This observation was made in a weakly immunogenic 3-methylcholanthrene-induced murine fibrosarcoma MCA 205 model and in a poorly immunogenic murine melanoma D5 model that are both syngeneic to B6 mice [2]. In a murine 4T1 model of breast cancer syngeneic to Balb/c mice, we reported that the transfer of LPS/anti-CD40- activated 4T1 TDLN B cells significantly reduced the induction of spontaneous 4T1 pulmonary metastases, and these effector B cells could directly kill 4T1 tumor cells [3]. Together, these studies demonstrated that transferred effector B cells can act independently in eliciting tumor regression *in vivo* in several murine tumor models syngeneic to hosts with different genetic backgrounds.

Interleukin 2 (IL-2) is a pleiotropic cytokine that stimulates T-cell proliferation; enhances NK cytolytic activity, induces the differentiation of Tregs, and causes activation- induced cell death [4, 5]. However, the effect of IL-2 on B lymphocytes is not well defined. In addition, CXCR4 is a chemokine receptor specific for stromal-derived-factor-1(SDF-1), and is also known as CXCL12, a molecule with strong chemoattractant properties for lymphocytes [6, 7]. Furthermore, a property of cytotoxic lymphocytes is their expression and release of powerful toxins, including the pore-forming protein perforin [8, 9]. While perforin is known to be a cytolytic protein found in the granules of cytotoxic T lymphocytes (CTLs) and natural killer cells [10, 11], its role in B cells is unknown. In this present study, we examined new mechanisms contributing to direct B cell-mediated antitumor immunity, including the impact of IL-2, the CXCR4/CXCL12 pathway and perforin in mediating tumor regression after the adoptive transfer of B effector cells.

## RESULTS

### Inhibition of pulmonary metastases by TDLN B cells is enhanced with IL-2 administration in adoptive immunotherapy

**Figure 1:**
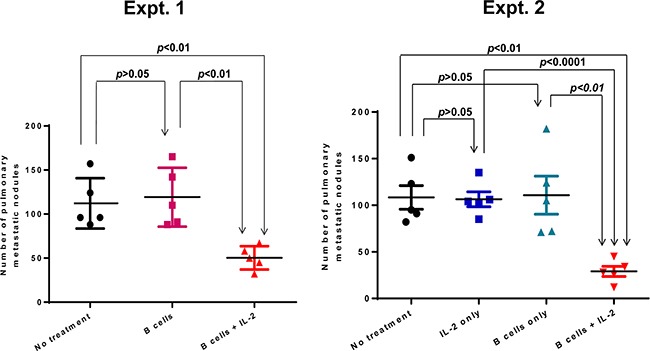
Adoptive transfer of a suboptimal does (1 × 10^6^) TDLN B cells plus IL-2 administration suppressed spontaneous pulmonary metastasis 4T1 TDLN B cells were adoptively transferred with or without IL-2 administration in mice with intramammary fat pad 4T1 tumors. After 2 weeks, the number of pulmonary metastases per mouse was enumerated. Each symbol represents an individual mouse. Two independent experiments are shown. Data are shown as mean ±SEM. p-values are indicated and determined by Student's t-test.

Although interleukin-2 was originally described as a “T cell growth factor”, we have found that it can significantly enhance the antitumor immunity of the B effector cells in adoptive therapy. In order to investigate the role of IL-2 in B cell-mediated adoptive immunotherapy, we examined the efficacy of transferred TDLN B cells given in a suboptimal dose (1 × 10^6^ cells/mouse) in conjunction with or without IL-2 administration. Fourteen days after 4T1 tumor cells were injected into the mammary fat pad, mice were administered with activated TDLN B cells alone or TDLN B cells plus IL-2. Fourteen days later, mice were euthanized to quantify pulmonary metastases. A suboptimal dose of B cells alone showed no efficacy, but B cells plus IL-2 administration i.p. significantly inhibited the induction of spontaneous pulmonary metastases (Figure 1, Expt. 1). However, IL-2 alone had no therapeutic effect compared to PBS-treated controls (Figure 1 Expt. 2). These experiments indicated that exogenous IL-2 administration augmented the therapeutic efficacy of transferred effector B cells.

**Figure 2:**
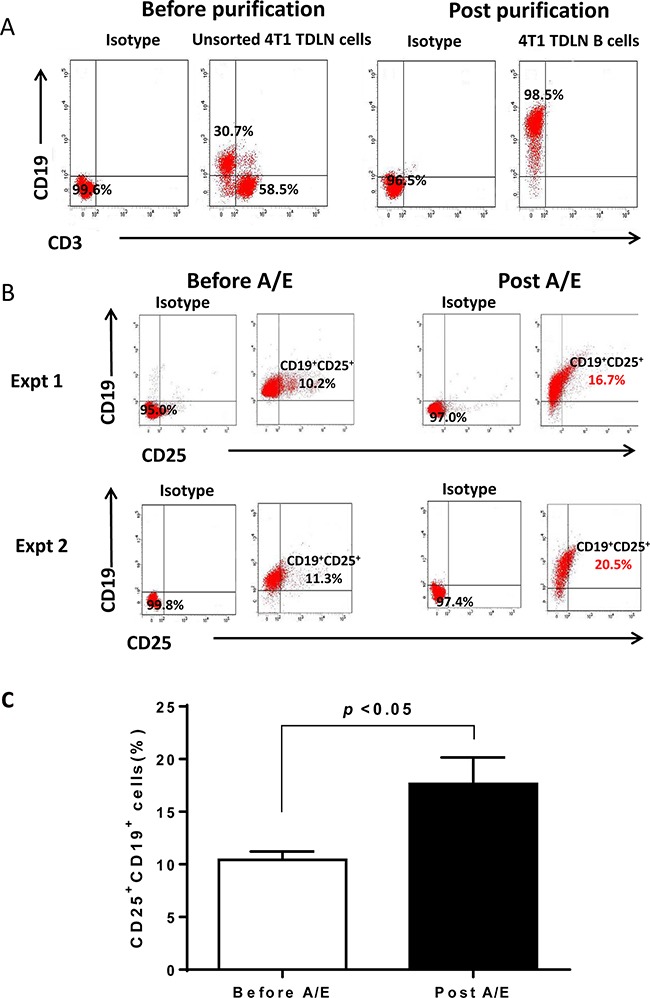
Phenotype of 4T1 TDLN B cells **A.** The purity of 4T1 TDLN B cells. TDLN cells were double stained with anti-CD3 and anti-CD19 before and after CD19^+^ cell purification. **B.** Detection of IL-2R (CD25) on the cell surface of purified 4T1 TDLN B cells. B cells were double stained with anti-CD19 and anti-CD25 antibodies before and after B cell activation and expansion (A/E). Results are shown in two independent experiments performed. **C.** 4T1 TDLN B cell activation and expansion (A/E) significantly augmented IL-2R(CD25)-expressing B cells.

In follow-up experiments, we investigated whether IL-2 receptor (IL-2R) was expressed on activated B effector cells. We purified TDLN B cells for this purpose. Unsorted 4T1 TDLN cells before purification are composed of approximately 30% CD19^+^ B cells and 60% CD3^+^ T cells, while cell purification enriched the CD19^+^ B cells to > 95% (Figure 2A). We then tested IL-2R (CD25) expression on the purified 4T1 TDLN B cells (Figure 2B). Before B cell activation and expansion, about 10% of the B cells expressed CD25. B cell activation and expansion almost doubled the number of B cells expressing CD25, resulting in almost 20% of the B cells expressing CD25. These results suggest that IL-2 most likely interacts with TDLN B cells by directly binding to IL-2R. Figure 2A represents the effective purification of B cells from TDLN cells, which we have reported in a few of our previous publications [2, 3, 12]. Repeated experiments shown in Figure 2B allowed us to generate a bar graph with p values, indicating that B cell activation and expansion (A/E) significantly (p<0.05) augmented the CD25 (IL-2R)-expressing B cells (Figure 2C).

### IL-2 modulates antitumor humoral responses in the host treated with adoptively transferred TDLN B cells

**Figure 3:**
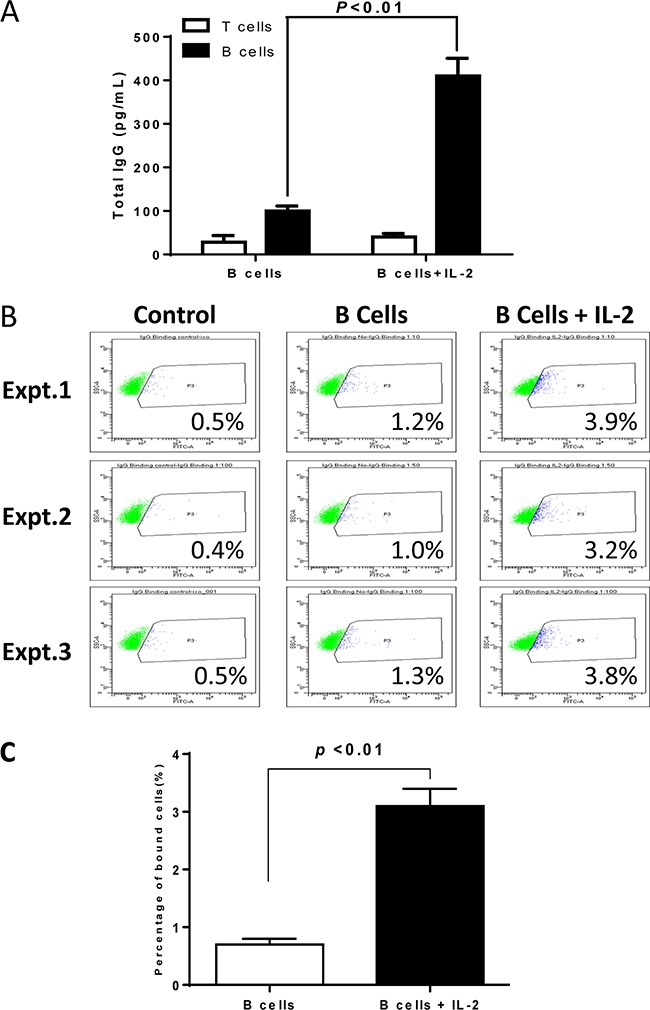
Humoral responses against 4T1 in the hosts subjected to 4T1 TDLN B cells + IL-2 therapy **A.** IgG production by host T and B cells purified from the spleens of animals subjected to TDLN B cell therapy with *vs.* without IL-2. **B.** Flow cytometry showing the binding effect of IgG to 4T1 tumor cells. IgG are from the culture supernatants of spleen B cells as prepared in Figure 3A. Binding experiments were repeated 3 times. **C.** Binding to 4T1 cells by IgG from culture supernatants of spleen B cells harvested from the 4T1 bearing mice treated with 4T1 TDLN B cells alone or 4T1 TDLN B cells plus IL-2 administration.

To determine the mechanism(s) that IL-2 enhances the antitumor immunity of 4T1 TDLN B effector cells in adoptive therapy of 4T1-bearing animals, we collected host spleens at the end of the treatments. Splenic T and B cells were enriched and subsequently activated *in vitro* with anti-CD3/anti-CD28 or LPS/anti-CD40 respectively. Lastly, culture supernatants were collected for antibody detection. While we found no antibody in T cell cultures as expected, we found significantly (p<0.01) higher IgG production by B cells isolated from animals treated with B cells + IL-2 compared with B cell alone treatment groups (Figure 3A).

To test the specificity of the antibody, we assessed the binding of the immune supernatants of cultured splenic B cells against 4T1 tumor cells. As shown in Figure 3B, in three replicate experiments, we observed that splenocytes from hosts subjected to B cell therapy plus IL-2 administration produced antibody which bound to 4T1 cells (3.2-3.9%) more than the antibody produced by splenocytes from hosts subjected to B cell therapy alone (1.0-1.3%), and was statistically significant (p<0.01, Figure 3C). These data demonstrated enhanced systemic humoral responses against 4T1 in the hosts treated with TDLN B cells + IL-2 therapy. While we did not analyze the isotype of the IgG in this study, we have previously reported that the major isotype was IgG2b [13]. This IgG2b binds specifically to relevant tumor cells. Similarly, the binding was low (from 1.4% to 5.4% in multiple assays), but this binding was tumor specific since their binding to an irrelevant tumor target was 0-0.3%. More importantly, such binding was immunologically significant because such binding resulted in complement dependent cytotoxicity [13].

### 4T1 TDLN B cell-mediated cytotoxicity of 4T1 tumor cells involves the CXCR4/CXCL12 pathway

**Figure 4:**
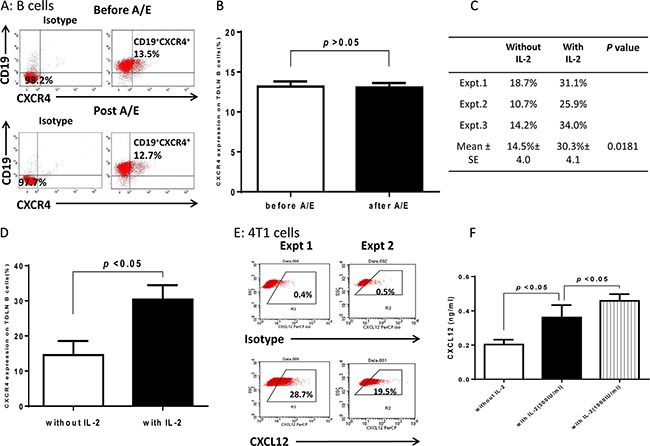
Expression of CXCR4 in 4T1 TDLN B cells and CXCL12 in 4T1 tumor cells **A.** Purified 4T1 TDLN B cells express CXCR4. B cells were prepared as in Figure 2 and double stained with PE-anti-CD19 and FITC-anti-CXCR4 before and after activation/expansion (A/E) respectively. **B.** CXCR4 expression on 4T1 TDLN B cells before and after A/E without IL-2. **C.** Comparison of CXCR4 expression on 4T1 TDLN B cells with *vs*. without IL-2. **D.** IL-2 significantly increased expression of CXCR4 on 4T1 TDLN B cells. **E.** 4T1 tumor cells express CXCL12. Data from two independent experiments of flow cytometry assays are shown. **F.** IL-2 significantly augmented CXCL12 production by cultured 4T1 cells as detected in the culture supernatant using ELISA.

Interaction of chemokine and chemokine receptors represents a key mechanism in the attraction and recruitment of immune cells to cancer cells [14, 15]. We examined the expression of several chemokine receptors on purified 4T1 TDLN B cells by flow cytometry, e.g. CXCR2, CXCR4, CCR5, CCR7, and CCR10. We found that CXCR2 and CCR7 were undetectable, and CCR5 or CCR10 expressed on <5% B cells (data not shown). However, CXCR4 expression was comparatively high on purified 4T1 TDLN B cells and continued to be high after LPS/anti-CD40 activation/expansion (12-15%, Figure 4A). Statistically, CXCR4 expression on B cells did not change significantly (p>0.05) after B cell activation and expansion (A/E) in the absence of IL-2 (Figure 4B). We then performed additional experiments comparing the CXCR4 expression on B cells in the presence or absence of IL-2. As shown in Figures 4C and 4D, CXCR4 expression was significantly increased when B cells were cultured with the addition of IL-2 (300 IU/ml). The culture conditions were the same as those used for Figure 4A. CXCR4 expression was 14-19% without the use of IL-2 (Figure 4C). However, in the presence of IL-2, CXCR4 expression was increased to 25-34%. The increased CXCR4 expression on B cells in the presence of IL-2 was statistically significant (p<0.05) compared with that in the absence of IL-2 (Figure 4D).

CXCR4 is the receptor for CXCL12 (SDF-1) which has been shown to mediate target cell chemotaxis by cells expressing CXCR4 [15]. We detected the expression of intracellular CXCL12 on 4T1 cells and found that approximately 20-30% of 4T1 cells expressed CXCL12 (Figure 4E, Expt 1 & 2). To provide more evidence that IL-2 augments the TDLN B cell killer/effector function via the CXCR4/CXCL12 axis, we performed experiments to compare the CXCL12 production by 4T1 tumor cells in the presence or absence of IL-2. In Figure 4F, we cultured 4T1 cells with various doses of IL-2, collected the culture supernatants, and tested the production of CXCL12 using ELISA. IL-2 significantly enhanced the production of CXCL12 by 4T1 cells in an IL-2 dose dependent manner.

**Figure 5:**
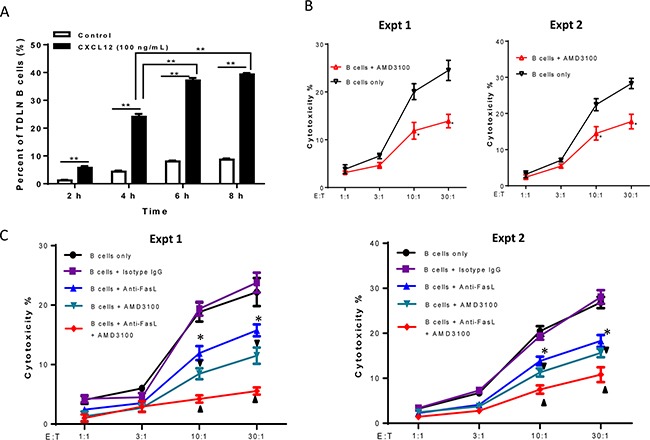
Involvement of CXCR4/CXCL12 pathway in 4T1 TDLN B cell-mediated cytotoxicity of 4T1 tumor cells **A.** The chemotaxis of activated 4T1 TDLN B cells detected by transwell assay. 4T1 TDLN B cells were put in the upper chamber of the transwell, while CXCL12 was added to the lower chamber of the transwell. After incubation for 2, 4, 6, and 8 hours, cells in the lower chamber were enumerated using a hemocytometer. ** p<0.01. The chemotactic index (%) represents the ratio of cells migrating in the presence of chemokines to the number of cells migrating in the absence of cytokines at each time point analyzed in triplicate. Results are shown as mean±SEM of triplicate counts from a single experiment representative of three experiments performed. **B.** CXCR4 specific inhibitor, AMD3100 (2.5 μg/ml), blocked cytotoxicity of 4T1 tumor cells mediated by effector 4T1 TDLN B cells in the LDH assays. *p<0.05, B cells + AMD3100 *vs.* B cells only at the ratios of 10:1 and 30:1 in two experiments performed. (**C**) Additively blocked cytotoxicity of 4T1 tumor cells mediated by 4T1 TDLN B cells with anti- FasL and AMD3100. The B cells were co-cultured with 4T1 cells with or without the addition of AMD3100 (2.5 μg/ml) and/or anti-FasL (10 μg/ml) in the LDH assays. Anti-FasL mAb isotype- matching IgG was used as control. *p<0.05, B cells + anti-FasL *vs.* B cells only or *vs.* B cells + isotype IgG; ▼ p<0.05, B cells + AMD3100 *vs.* B cells only or *vs.* B cells + isotype IgG; ▲ p<0.05, B cells + anti-FasL + AMD3100 *vs.* B cells + anti-FasL or *vs.* B cells + AMD3100. Data are generated from two independent experiments as shown.

To ascertain the chemotactic effects of CXCL12 on TDLN B cell migration, transwell assays were performed. TDLN B cells were purified and used for the chemotaxis assays as described in the Methods section. Compared with the control group (complete medium supplemented with 10% FBS) at each time point, the addition of CXCL12 to the medium in the lower chambers resulted in significant (p < 0.01) migration of TDLN B cells from the upper chamber to the lower chamber with time (Figure 5A).

Righi et al. have previously shown that AMD3100, a highly specific CXCR4 antagonist, blocks the binding of CXCR4 to CXCL12 [16]. We added AMD3100 to block the interaction of CXCL12 with CXCR4 during the LDH release assay while testing B cell cytotoxicity against 4T1 tumor cells. For this assay, effector B cells were generated from 4T1 TDLN B cells as described in the Methods section. Target cells (4T1) were plated with B cells at various effector to target cell ratios with or without the addition of AMD3100 (2.5 μg/ml) as indicated in Figure 5B. AMD3100 significantly (p<0.05) inhibited B cell-mediated 4T1 tumor cell death at the E:T ratios of 10:1 and 30:1 in 2 of 2 experiments performed. These results suggest that the interaction of CXCR4 on B cells and its ligand CXCL12 produced by 4T1 cells is involved in the direct cytotoxicity of 4T1 cells by B cells.

We have recently reported that TDLN B cells directly kill 4T1 tumor cells via the Fas/FasL pathway [12]. To examine whether B cell-mediated 4T1 tumor cell death involves both Fas/FasL and CXCR4/CXCL12 pathways, we added anti-FasL and AMD3100 in the same experiment. As shown in Figure 5C, anti-FasL alone significantly (p<0.05) suppressed the killing of activated B cells on 4T1 tumor cells at different E:T ratios; AMD3100 alone also significantly (p<0.05) inhibited such killing, which was consistent with our observations in the previous experiments. Of note, anti-FasL and AMD3100, when used simultaneously, resulted in significantly greater inhibition of the 4T1 tumor cell cytotoxicity by 4T1 TDLN B cells compared to anti-FasL or AMD3100 alone (p<0.05). These results support the conclusion that the cytotoxicity of 4T1 tumor cells by effector B cells involves both Fas/FasL and CXCR4/CXCL12 pathways.

### The role of perforin in B cell-mediated cytotoxicity of tumor cells

**Figure 6:**
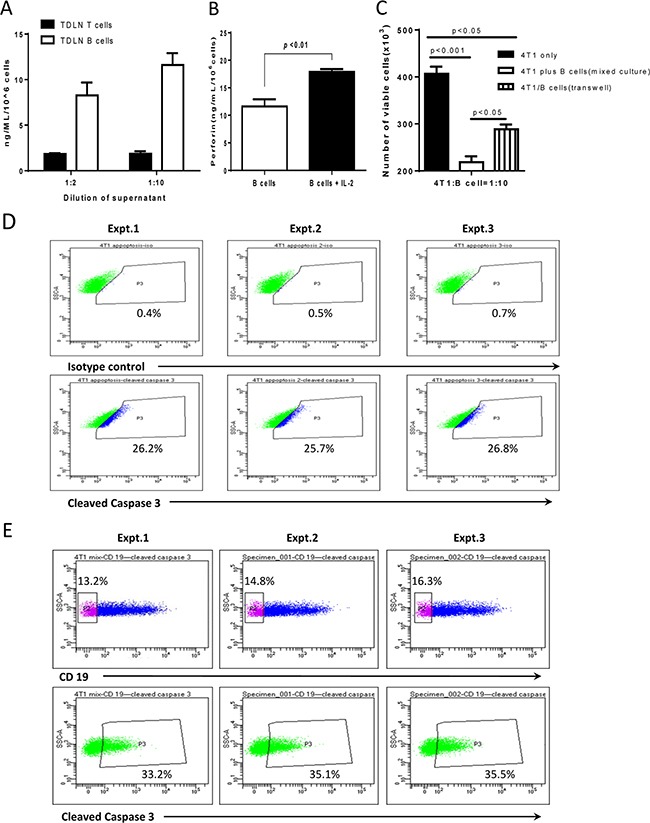
Perforin in 4T1 TDLN B cell-mediated cytotoxicity of 4T1 cells **A.** TDLN B cells produce perforin. Supernatant was collected from cultured 4T1 TDLN T cells and B cells respectively, and was detected for perforin production by ELISA. The dilution of the supernatant was 1:2 and 1:10 as indicated. Results are shown as mean ± SEM of triplicate wells from a single experiment representative of three experiments performed. **B.** IL-2 significantly enhanced perforin production by 4T1 TDLN B cells as detected in the B cell culture supernatant using ELISA. **C.** The cytotoxity of activated 4T1 TDLN B cells against 4T1 cells was detected by mixed cells and in transwell respectively. The ratio of 4T1 cells : B cells was 1:10. The starting 4T1 cell number was 400, but was significantly (p<0.001) decreased in the mixed culture with 4T1 and B cell contact as well as in the transwell experiment without 4T1 and B cell contact (p<0.05). However, there was a significant (p<0.05) difference between these two groups: mixed-culture *vs.* transwell. **D.** In transwell assays, after 6 hours incubation, 4T1 cells in the lower chamber were fixed and permeabilizated, followed by staining with anti-mouse cleaved caspase-3 mAb. **E.** Cell-cell contact resulted in more 4T1 cells expressing cleaved caspase-3 than those in the transwell. In cell-cell contact assays, mixed B cells and 4T1 cells were harvested after incubation for 6 hours, surface stained with anti-CD19, and then fixed and permeabilizated, followed by staining with anti-mouse cleaved caspase-3 mAb. The 4T1 cells were gated from the CD19 negative population. Data of three experiments are shown.

Collectively, the above experiments demonstrated that B cells could directly kill tumor cells via cell-cell contact. It is well known that perforin, a small secreted pore-forming granule protein, can result in cell death after it is introduced into the target cell [17, 18]. To examine the potential role of perforin in B cell-mediated cytotoxicity of 4T1 cells, we collected 4T1 TDLN T cells and B cells, and activated T cells with anti-CD3/anti-CD28 and B cells with LPS/anti-CD40 respectively as described in the Methods section. We collected the culture supernatants and detected perforin produced by T cells and B cells (Figure 6A). Figure 6A indicates that perforin is released not only by T cells but also by the activated TDLN B cells. To provide experimental evidence that IL-2 augments the TDLN B cell killer/effector function via the perforin pathway, we performed experiments to compare the perforin production by 4T1 TDLN B cells in the presence and absence of IL-2. As shown in Figure 6B, addition of IL-2 to the B cell culture significantly (p<0.01) increased the production of perforin. To further define the cytotoxic mechanisms of B cells, we compared B cell-mediated cytotoxicity of 4T1 cells with direct cell contact (i.e. mixed cell cultures) vs. no cell contact (i.e. transwell cultures) (Figure 6C). The starting 4T1 cell number was 400 as indicated. At the 4T1: B cell ratio = 1:10, about 50% of the 4T1 cells (approximately 200 cells) were killed by B cells in mixed culture. However, in the transwell experiment, about 75% (approximately 300 cells) survived, and about 25% of the 4T1 cells (approximately 100 cells) were killed. These experiments thus suggest that soluble factors, such as perforin, could lead to partial lysis of 4T1 cells as shown in the transwell experiment, but cell contact is needed for optimal B cell-mediated cytotoxicity of 4T1 cells as shown in the mixed culture experiment.

To better assess the cell death of 4T1 cells, we examined one of the cell death pathway molecules, cleaved caspase 3, by flow cytometry to verify the data assessed by trypan blue in Figure 6C. Figure 6D shows that, in transwell experiments as described in Figure 6C, cleaved caspase 3- positive cells was 25%-27% of the total 4T1 cells in 3 of 3 independent experiments performed. Furthermore, in the cell-cell contact assay, also as described in Figures 6C and Figure 6E shows that 13-17% of the mixed cells were CD19^−^, e.g. 4T1 cells. More 4T1 cells expressed cleaved caspase-3 (33-36% of the total 4T1 cells) than those 4T1 cells in the transwell experiments as shown in Figure 6D.

**Figure 7:**
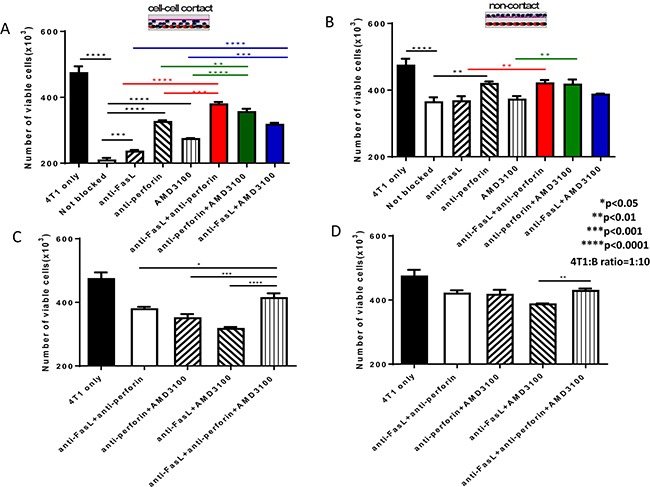
Cytotoxicity of 4T1 tumor cells mediated by 4T1 TDLN B cells involves Fas/FasL and CXCR4/CXCL12 pathways as well as perforin **A.** B cell killing of 4T1 tumor cells in cell-cell contact in the presence of anti-FasL, anti-perforin and AMD3100 either by each itself or by any 2 of the 3 in combination. **B.** B cell killing of 4T1 tumor cells without cell contact in the presence of anti-FasL, anti-perforin and AMD3100 either by each itself or by any 2 of the 3 in combination. **C.** B cell killing of 4T1 tumor cells in cell-cell contact in the presence of anti- FasL+anti-perforin +AMD3100 compared with the use of any 2 of the 3 in combination. **D.** B cell killing of 4T1 tumor cells without cell contact in the presence of anti-FasL+anti-perforin +AMD3100 compared with the use of any 2 of the 3 in combination. Results are shown as mean±SEM of triplicate wells from a single experiment representative of three experiments performed.* p<0.05, ** P<0.01, *** P<0.001, **** P<0.0001.

In Figure 5, we measured the cytotoxicity of 4T1 tumor cells by B cells in the presence of anti-FasL and the CXCR4-specific inhibitor AMD3100, and found that both Fas/FasL and CXCR4/CXCL12 pathways are involved in B cell-mediated killing of tumor cells. To examine the role that perforin may have, we performed additional transwell experiments in the presence of anti-FasL, AMD3100 and anti-perforin at the same time. Based on Figure 5C, anti-FasL demonstrated a very specific reactivity as its isotype IgG control showed no non-specific effects in 2 of the 2 experiments performed. In addition, Lehmann et al described a very specific inhibitory effect of anti-perforin antibody on granule-mediated killing of K562 cells when an isotype control antibody was used [19]. B cells were co-cultured with 4T1 at the ratios of 10:1 in transwells as described in the Methods section. Anti-FasL, AMD3100 and anti-perforin were added either separately or together as indicated in Figure 7. After 12h, viable 4T1 cells were counted in the lower chambers. As shown in Figure 7A, when B cells and tumor cells were co-cultured with cell-cell contact, anti- FasL, anti-perforin and AMD3100 alone each blocked the 4T1 cell killing significantly compared with the control (not blocked). In contrast, without cell contact (Figure 7B), only anti-perforin alone blocked the 4T1 cell killing significantly compared with the control (not blocked). In combination, anti-FasL+anti-perforin; anti-perforin+AMD3100; and anti-FasL+AMD3100 blocked the 4T1 cell killing significantly compared with the use of each blocking agent alone with cell-cell contact (Figure 7A). By contrast, without cell contact (Figure 7B), while anti-FasL+anti-perforin and anti- perforin+AMD3100 blocked the 4T1 cell killing significantly compared with anti-FasL or AMD3100 alone, anti-FasL+AMD3100 demonstrated no significant difference in blocking the 4T1 cell killing compared with the use of anti-FasL or AMD3100 alone. Finally, the anti-FasL+anti-perforin+AMD3100 triple-blocked group showed significantly increased blocking effect on 4T1 cell killing compared with the double blocking using any 2 of the 3 combinations in the cell-cell contact assays (Figure 7C). However, without cell contact (Figure 7D), this triple-blocked group revealed significantly increased blocking effect on 4T1 cell killing only when compared with the double blocking using anti-FasL+AMD3100. Collectively, these results indicate that effector B cells directly kill tumor cells in cell-cell contact via the Fas/FasL and CXCR4/CXCL12 pathways as well as perforin, while without cell contact, perforin secreted by B cells could also lead to tumor cell cytotoxicity.

## DISCUSSION

We previously reported that purified TDLN B effector cells could kill tumor cells directly involving the Fas/FasL pathway and are regulated by IL-10 [12]. Like T cells, cytotoxic B cells may need to modulate multiple signaling pathways and are regulated by multiple cytokine/chemokines to mediate optimal cytotoxicity against tumor cells. In the current study, we further defined the mechanisms involved in B cell-mediated killing of tumor cells and found that activated B effector cells killed tumor involving the CXCR4/CXCL12 and perforin pathways as well as the Fas/FasL interaction, and can be augmented by IL-2.

**Figure 8:**
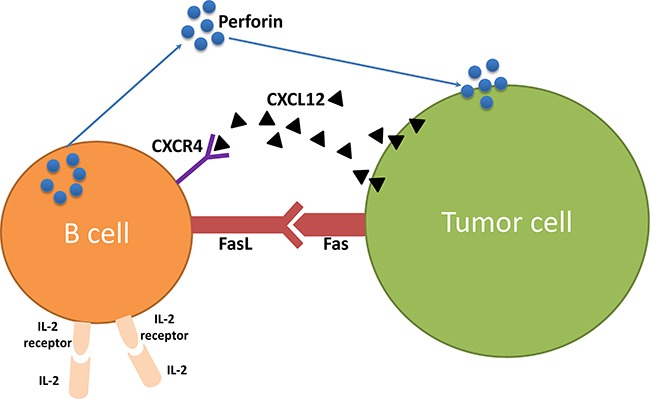
The role of IL-2 in B cell anti-tumor immunity

Our data revealed that IL-2 can enhance B cell killer/effector function. It has been well documented that T cells from tumor or tumor draining lymph nodes, after activation *in vitro*, are effective in cancer immunotherapy and could kill tumor cells more effectively *in vivo* in combination with IL-2 administration [1, 3, 13, 20, 21]. In this study, we found that effector B cells also express IL-2R and that exogenous IL-2 administration significantly augmented the suppression of 4T1 lung metastasis. These data demonstrate for the first time that IL-2, can also serve as an adjuvant to adoptively transferred TDLN B cells *in vivo* to enhance their antitumor immunity. In this study, we have shown that B cells express IL-2R (CD25). Our *in vitro* experiments demonstrated that IL-2 modulated expression of CD25 on B cells after activation /expansion (A/E) before adoptive transfer; enhanced CXCR4 expression on B cells; augmented CXCL12 production by 4T1 tumor cells, and increased production of perforin by B cells. In addition, we found significantly more binding to 4T1 tumor cells by IgG containing culture supernatants of splenic B cells collected from the hosts subjected to B cell therapy plus IL-2 administration vs. B cell therapy only. Collectively, these data indicate the direct effects of IL-2 on B cells. As a corollary, IL-2 administration in mice may stimulate CD4^+^ T cell proliferation in tumor-bearing mice, and it is well-known that the Th2 cells can provide help for B cell function [22, 23]. In this report, we would like to focus on our new findings that IL-2 may act upon B cells directly. Figure 8 represents a summary diagram showing the role of IL-2 in B cell anti-tumor immunity.

We have previously reported that activated TDLN B cells mediate tumor cytotoxicity, the Fas/FasL pathway [12]. In the current study, we investigated other mechanisms by which activated B cells kill tumor cells. The CXCR4/CXCL12 axis represents a major mechanism in tumor growth and metastasis, and its role in the cancer cell-tumor microenvironment has recently been studied regarding cancer progression and the attraction/activation of leukocytes [14, 24, 25]. While it was reported that CXCR4 may be expressed on both lymphocytes and cancer cells [15], we found that 4T1 TDLN B cells expressed CXCR4. In addition, the 4T1 tumor cells overexpress CXCL12. Functionally, we found that CXCR4 blockade using a CXCR4-specific inhibitor, AMD3100, significantly blocked B cell-mediated killing of 4T1 tumor. Of note, blockade of FasL and CXCR4 simultaneously inhibited B cell-mediated killing of tumor cells in an additive manner, indicating that both Fas/FasL and CXCR4/CXCL12 axes are involved in the killing of tumor cells by B cells.

Perforin is a cytolytic protein that binds to the tumor cell plasma membrane to form pores leading to its destruction [26, 27]. Perforin is found in cytotoxic T cells and natural killer cells. In this study, we found that effector B cells release perforin which can mediate tumor cell killing. Our study also found that effector B cells can kill tumor cells via cell-cell contact utilizing the Fas/FasL and CXCR4/CXCL12 pathways as well as perforin, while without cell contact, perforin secreted by B cells can also lead to tumor cell cytotoxicity. While perforin has been extensively studied in T cells, no previous report has described its role in B cell-mediated cell killing. In addition, we demonstrated that IL-2 could significantly (p<0.01) enhance perforin production by activated B cells. Together, these data support our conclusion that IL-2 modulates several pathways contributing to the killing of tumor cells by B effector cells.

In summary, exogenous IL-2 administration significantly enhanced the antitumor reactivity of adoptively transferred effector B cells. Purified B effector cells kill tumor cells directly and such killing involves not only the Fas/FasL pathway, but also the CXCR4/CXCL12 pathway and perforin. These studies further identify the mechanisms by which effector B cells mediate antitumor reactivity which can be applied to future cancer immunotherapies.

## MATERIALS AND METHODS

### Mice

Female BALB/c mice were purchased from the Jackson Laboratories, Bar Harbor, ME. They were maintained in a pathogen-free environment and used at age 7 weeks or older. Principles of laboratory animal care (NIH publication No. 85-23, revised 1985) were followed. The animal protocols were approved by the University of Michigan Laboratory of Animal Medicine.

### Murine tumor cells

The 4T1 cell line is a mammary carcinoma syngeneic to BALB/c mice (kindly provided by Dr. M. Sabel, University of Michigan). Inoculating 4T1 cells into the mammary fat pad induces the development of spontaneous pulmonary metastases. 4T1 cells were maintained *in vitro* in complete medium (CM).

### Tumor draining lymph nodes (TDLNs)

In order to induce TDLNs, 1 × 10^6^ 4T1 tumor cells in 0.1 ml PBS were injected subcutaneously (s.c.) into the lower flanks of BALB/c mice. Nine days after 4T1 cell inoculation, the draining inguinal lymph nodes were collected. The TDLNs were processed as previously described [12].

### T cell and B cell activation and expansion

CD19^+^ B cells were purified from the TDLN cells or splenocytes using anti-CD19- coupled microbeads and the MACS separator (MiltenyiBiotec. Inc. Auburn, CA). CD3^+^ T cells were purified from the TDLN or splenocytes using anti-CD3-coupled microbeads. B cells were activated with lipopolysaccharide (LPS, Sigma-Aldrich, Atlanta, GA) plus anti-CD40 (FGK45) mAb ascites in complete medium at 37°C with 5% CO_2_ for 3-4 days. T cells were activated with immobilized anti-CD3 and anti-CD28 mAbs in CM containing IL-2 (Prometheus Laboratories, San Diego, CA). The culture supernatants were collected and stored at −20°C for future experiments.

### Flow cytometry analysis

Cell surface expression of CD19, CD25 and CXCR4 and intracellular expression of CXCL12 and cleaved caspase-3 were analyzed by immunofluorescence assay. All fluorescein isothiocyanate (FITC)-or phycoerythrin (PE) -or peridinin chlorophyll protein (PerCP) - conjugated antibodies (FITC rat anti-mouse CD19, PE rat anti-mouse CD19, PE rat anti-mouse CD25, FITC rat anti-mouse CXCR4, and PerCP rat anti-mouse CXCL12) and matched isotype controls were purchased from BD Biosciences (San Jose, CA). Alexa Fluor® 488-conjugated Rabbit anti-mouse/human cleaved caspase-3 antibody and matched control were purchased from R&D system (Minneapolis, MN). To measure intracellular CXCL12 expression, 1 million 4T1 cells were incubated with 2 μl/ml Leukocyte Activation Cocktail (PMA/ionomycin/Golgiplug, BD Pharmingen, San Jose, CA) and 0.67 μl/ml Golgistop (BD Pharmingen, San Jose, CA) in 6- well plate for 4-6 hours at 37°C with 5% CO_2_. After fixation and permeabilization with Fixation/Perm Buffer (eBioscience, Inc., San Diego, CA), the cells were stained with PerCP rat anti-mouse CXCL12 mAb. In transwell assays, after 6 hours, 4T1 cells in the lower chamber were fixed and permeabilizated, followed by staining with anti-mouse cleaved caspase-3 mAb. In cell-cell contact assays, mixed B cells and 4T1 cells were harvested after incubation for 6 hours, surface stained with anti-CD19, and then fixed and permeabilizated by eBioscience Fixation and Permeabilization fixed kit, followed by staining with anti-mouse cleaved caspase-3 mAb. The 4T1 cells were gated from the CD19 negative population. Flow cytometry was performed on a LSRII flow cytometer (BD Biosciences). BD FACSDiva software (version 7.0) was used for all flow cytometry analysis.

### Adoptive TDLN B cell therapy of 4T1 cancer

Healthy Balb/c mice were inoculated with 5×10^4^ 4T1 cells into the mammary fat pad to induce spontaneous pulmonary metastases. Fourteen days after tumor inoculation, the tumor- bearing mice were treated with tail vein injection of activated 4T1 TDLN B cells. Commencing on the day of the effector B cell transfer, intraperitoneal (i.p.) injections of IL-2 (40,000 IU) (Prometheus Laboratories, San Diego, CA) were administered in 0.5 ml of PBS and continued twice daily for 8 doses. About 2 weeks after B cell transfer, all mice were sacrificed and lungs were harvested for enumeration of spontaneous pulmonary metastatic nodules. At the same time, spleens were collected for purification of splenic T and B cells as described above.

For 4T1 TDLN B cell cytotoxicity in the presence of anti-FasL antibody (Biolegend Inc., San Diego, CA) and/or AMD3100 (Sigma, Atlanta, GA), the effector B cells were generated as described above and cultured with 4T1 cells with the admixture of 20 μg /ml anti-FasL and/or 2.5 μg /ml AMD3100 to block FasL and/or CXCR4; respectively.

### IgG binding by immune supernatant

4T1 cells were incubated with the immune supernatants collected from the cultured B cells with equal quantities of IgG followed by incubation with the 2^nd^ antibody: FITC-conjugated anti-mouse IgG. The concentration of total IgG was measured by ELISA quantitation kits (Bethyl laboratories, Montgomery, TX). The binding of supernatant antibody to 4T1 cells was assessed using flow cytometry.

### Chemotaxis assay

In transwell experiments, 5×10^5^ 4T1 TDLN B cells in 200 μl were added to the upper chamber of a transwell (insert pore size, 5 μm; Millipore, Billerica, MA). CXCL12 (100 ng/ml, R&D Systems, Minneapolis, MN) was added to the lower chamber in a volume of 900 μl. After 2, 4, 6, and 8h of incubation at 37°C, cells that migrated into the lower chamber were enumerated using a hemocytometer. The chemotactic index (%) represents the ratio of cells migrating in the presence of chemokines to the number of cells migrating in the absence of cytokines at each time point analyzed in triplicate.

### LDH cytotoxicity assay

Cell cytotoxicity was assessed by measuring the release of cytoplasmic lactate dehydrogenase (LDH) into cell culture supernatants according to the manufacturer's protocol (CytoTox 96 Non-Radioactive Cytotoxicity Assay, Promega, Madison, WI). For TDLN B cell cytotoxicity, effector B cells were generated from 4T1 TDLN B cells using LPS/anti-CD40 as described above. Target cells were plated in triplicates in a 96-well U-bottom tissue culture plate (5000 cells/well) and co-incubated with TDLN B cells at effector to target cell ratios of 1:1, 3:1, 10:1 and 30:1. After 12 hours of incubation, cells were centrifuged and 50 μl supernatant from each well was transferred to a fresh 96-well plate, 50 μl of the substrate mix was added and incubated at room temperature in the dark for 15 to 30 min. Before LDH measurement, 50 μl of stop solution was added to each well. Maximal release of LDH was performed by incubating the target cells with Lysis Solution (Promega, Madison, WI). Target cells without effector cells were used as a spontaneous release control. Absorbance was measured at 490 nm using a 96-well plate reader.

### Perforin release assessment

T cells and B cells purified from 4T1 TDLNs were activated as described above. Culture supernatants were then collected for perforin release detection using ELISA kits (Cloud-Clone Corp, Houston, TX).

### The cytotoxity and blockage assay

In cell-cell contact assays, 4T1 cells were plated in triplicates in a 12-well tissue culture plate (1×10^5^ cells/well, 1ml) and co-incubated with 4T1 TDLN B cells (1×10^6^ cells/well, 1ml). In 0.4 μm transwell assay, 1×10^6^ 4T1 TDLN B cells were added to the upper chamber of a transwell, and 1×10^5^ 4T1 cells were added to the lower chamber. Anti-FasL(20μg/ml), anti-perforin (5μg/ml) and AMD3100 (2.5μg/ml) were added either alone or in combination as indicated in the experiments. After 12 hours of incubation, trypan blue staining was used to count the viable 4T1 cells either in the mixed co-culture or in the lower chamber of the transwell. The suspended B cells in the medium of the lower chambers were removed, and the well was washed by PBS for 3 times. B cells were distinguished from 4T1 cells by their significant different morphological characteristics under the microscope.

### Statistical analysis

Statistics were analyzed using the mean ± standard error. The significance of differences in numbers of metastatic nodules and cell lysis was determined using Student's t-test. *P*<0.05 was considered statistically significant between the experimental groups. For comparison of more than 2 groups, statistical significance was determined using a one-way ANOVA followed by a Bonferroni multiple-group comparison test. Statistical analyses were performed with Graph- Pad Prism 6.0.

## Abbreviations

TDLN: tumor-draining lymph node
CM: complete medium
s.c.: subcutaneous
i.v.: intravenously
i.p.: intraperitoneal
